# Longitudinal Curricular Assessment of Knowledge and Awareness of Intimate Partner Violence among First-Year Dental Students

**DOI:** 10.3390/ijerph18116039

**Published:** 2021-06-04

**Authors:** Charles Buchanan, Karl Kingsley, Rhonda J. Everett

**Affiliations:** 1 Department of Clinical Sciences, Las Vegas—School of Dental Medicine, University of Nevada, 1700 W. Charleston Blvd, Las Vegas, NV 89106, USA; buchac5@unlv.nevada.edu (C.B.); rhonda.everett@unlv.edu (R.J.E.); 2 Department of Biomedical Sciences, Las Vegas—School of Dental Medicine, University of Nevada, 1001 Shadow Lane, Las Vegas, NV 89106, USA; 3 Health Sciences Center, Woody L. Hunt School of Dental Medicine, Texas Tech University, 5001 El Paso Drive, El Paso, TX 79905, USA

**Keywords:** intimate partner violence (IPV), domestic violence (DV), dental student survey

## Abstract

Background: Intimate partner violence (IPV) has previously been recognized as a major public health issue. Oral healthcare providers, such as dentists, are crucial to the screening and identifying of individuals experiencing IPV, since most injuries occur in the head and neck region. A lack of knowledge and awareness regarding teaching and learning about IPV in dental school curricula has been identified. Based upon the overall lack of knowledge, the objective of this study was to conduct a longitudinal assessment of knowledge, awareness, and beliefs regarding IPV among dental students in their first year of education. Methods: All students (*n* = 245) from three consecutive, first-year dental student cohorts (*n* = 81, *n* = 82, *n* = 82) were provided a brief and voluntary in-class survey in conjunction with an instructional workshop. The survey included questions designed to ascertain knowledge, awareness, and beliefs regarding IPV as a healthcare and dental issue before and after the instructional session. Differences in responses to the questions before and after the IPV educational workshop were measured using paired t-tests. Results: A total of *n* = 232 completed pre- and post-responses were received from all three first-year dental student cohorts (*n* = 76, *n* = 80, *n* = 76), representing an overall 94.6% response rate. Analysis of these data showed that the student population was predominantly male and white (non-minority), aged in their mid- to late twenties, with most students reporting no previous IPV education. The few students reporting previous IPV education were mainly younger (<25 years), which may represent more recent endeavors to increase awareness of IPV among students currently attending colleges and universities. Conclusions: The results of this study may suggest that information-specific seminars within the curriculum might be adequate to provide dental students with awareness and knowledge of IPV and specific information regarding local resources and referrals for any patients experiencing IPV.

## 1. Introduction

Intimate partner violence (IPV) has previously been recognized as a major public health issue among dental and oral healthcare professionals [1,2]. This has led to calls for increased data regarding awareness and knowledge among dental professionals to recognize and address deficiencies in training and surveillance for signs of IPV in clinical practice [3,4]. Increased knowledge and awareness of IPV among dental professionals has been demonstrated to significantly improve referrals for IPV-specific support services and other effective intervention programs [5,6].

Oral health care providers, such as dentists, are crucial to the screening and identifying of individuals experiencing IPV, since most injuries occur in the head and neck region [7,8]. The most commonly reported IPV traumas involve facial contusions and lacerations, dental concussion, and mandibular fractures [9,10]. However, only a few select reports have assessed awareness and knowledge among dental students regarding IPV recognition and the appropriate resources and referrals needed to implement IPV curricular integration [11,12].

These efforts may contribute, in part, to a larger focus on the development and implementation of IPV prevention programs that are being created in different and varied settings to address these important issues [13,14]. For example, community-based programs have been developed that demonstrate significant progress may be possible with significant input and feedback from victims, abusers, as well as the healthcare professional providers and prevention teams [15,16]. In addition, these efforts have found models among refugee and humanitarian missions that clearly demonstrate these types of approaches may provide significant benefits and might be well placed for integration into medical and healthcare education and curricula to improve knowledge, awareness, and willingness to engage in these critical areas [17,18,19].

As other medical and healthcare disciplines move towards comprehensive curricular reform to include domestic and interpersonal violence, the need for more information regarding awareness and knowledge among dental students becomes crucial for dental school administrators interested in these specific curricular reform efforts [20,21]. As evidence continues to emerge regarding misinformation and misconceptions about IPV among other healthcare students, the need to accurately assess and evaluate knowledge among dental students becomes critically important [22,23]. Based upon the overall lack of knowledge in this area, the objective of this study was to conduct a longitudinal assessment of knowledge, awareness, and beliefs regarding IPV among dental students in their first year of education.

## 2. Materials and Methods

### 2.1. Study Approval

The protocol for this study was reviewed and deemed Exempt by the Office for the Protection of Research Subjects (OPRS) and the Institutional Review Board (IRB) at the University of Nevada, Las Vegas (UNLV), under protocol “Retrospective Investigation of Course Content Evaluation by Students: A Survey of Domestic Violence Education and Experience among UNLV-SDM Dental Students” (OPRS#1103-3752M). Informed Consent was waived pursuant to the exemption under the Basic HHS Policy for Protection of Human Research due to Subjects, (46.101) Subpart A (b) regarding IRB exemption for research involving the use of education tests (cognitive, diagnostic, aptitude, achievement), in which the subjects cannot be identified directly or through identifiers.

In brief, students (*n* = 245) among three consecutive, first-year dental student cohorts (*n* = 81, *n* = 82, *n* = 82) were provided a brief and voluntary in-class survey, in conjunction with an instructional workshop, as part of the normal instructional curriculum in Treatment Planning and Diagnosis. The survey included questions designed to ascertain knowledge, awareness, and beliefs regarding IPV as a healthcare and dental issue before and after the instructional session. Inclusion criteria included students enrolled in the DS1 student curriculum and the exclusion criteria included any student that chose not to participate. The workshop was facilitated by the UNLV Student Wellness Center’s IPV/Domestic Violence prevention and outreach coordinator, a certified provider of policies, procedures, and information for all UNLV departments, faculty, staff, and students. The workshop was originally developed and organized through a collaborative effort between the UNLV Jean Nidetch Women’s Center, the Office of Student Conduct, Counseling and Psychological Services, the Office of Civic Engagement and Diversity, and the Multicultural Center, and was supported by a grant from the Office on Violence Against Women, US Department of Justice (Grant 1009-WA-AX-0022).

### 2.2. Educational Objectives

The objective of the workshop was to provide an introduction and overview of interpersonal and domestic violence (DV), followed by step-by-step directions for what a student, faculty, or staff member can do when someone (patient, student, faculty, or staff) discloses IPV or DV. This included on- and off-campus resources for victims and survivors of IPV/DV and relevant UNLV regulations, as well as Nevada Revised Statutes (NRS) code law for reporting IPV/DV. The pre-and post-survey was a brief questionnaire that addressed IPV awareness, resources, professional beliefs, and responsibilities, and personal IPV education and intervention beliefs, which had been previously used and validated [11,24,25].

### 2.3. Pre- and Post-Surveys

The pre- and post-surveys were physically attached to one another and were distributed at the beginning of the session prior to the commencement of the workshop. Surveys were color coded to ensure the students that chose to participate were completing the “pre” survey at the appropriate time. Once the workshop was completed, students were asked to complete the “post” survey and return all items for analysis. Each survey (pre- and post-) was assigned a numerical, non-duplicated identifier to prevent disclosure (and ensure confidentiality) of survey participants. Basic demographic information, such as age, race, and sex were included in the survey at the end of the post survey questionnaire.

### 2.4. Data Analysis

All responses and demographic information were manually transcribed into an Excel spreadsheet (Microsoft Excel for Microsoft 365, Version 2104; Redmond, WA, USA) for subsequent analysis. Demographic analysis of the study participants was summarized and presented as descriptive statistics. Differences between the study participants and the overall cohort demographics were analyzed using Chi square statistics, which are appropriate for categorical (demographic) data analysis. Differences in responses to the questions before and after the IPV educational workshop were measured using paired t-tests, which are appropriate for measuring changes in parametric data analysis.

### 2.5. Study Participants

All students enrolled in three consecutive dental student cohorts (*n* = 245) were asked to participate in an in-class voluntary questionnaire that was administered before and after an educational session specifically targeted towards IPV (Table 1). A total of *n* = 242 students were present in class on the days when the educational session in each of the cohorts was presented, with a total of *n* = 232/245 or 94.6% of the student participants completing the survey. The demographic analysis of the study participants was approximately one third females (36.6%) and two thirds males (62.5%), with no significant differences between the reported sex of the study participants and overall cohort demographics, *p* = 0.8368. Similarly, the majority of the study participants reported their race/ethnicity as non-minority or white (50.4%), which was similar to the overall demographics of the three cohorts (56.6%), *p* = 0.2268.

## 3. Results

**Figure 1:**
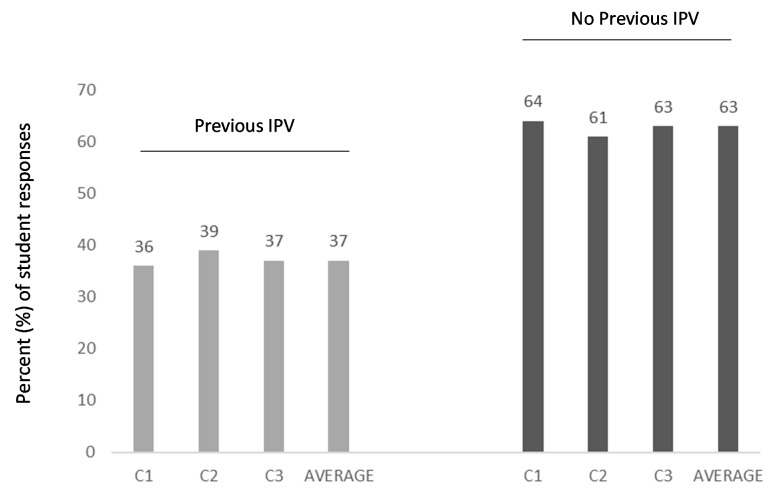
Previous IPV education among respondents. Approximately two-thirds (64%) of respondents indicated that they had no formal educational experience with either IPV or DV in a curricular setting or educational environment, with no significant changes over time during the time period evaluated by this study, *p* = 0.411. In addition, slight differences between males and females were noted, with a slightly higher percentages of males (71.1%) reporting no previous IPV or DV experience than their overall percentage in the school population (62.4%), which was not statistically significant, *p* = 0.1229.

The first survey question sought to evaluate knowledge and awareness among these dental students by asking whether they had previous educational experience with IPV or DV (Figure 1). More specifically, approximately two-thirds (64%) of respondents indicated that they had no formal educational experience with either IPV or DV in a curricular setting or educational environment. As many changes to educational systems and curricular interventions occurred over time, the data from each cohort were evaluated independently, which demonstrated that there were no significant changes over time during the time period evaluated by this study, *p* = 0.411. In addition, slight differences between males and females were noted, with a slightly higher percentages of males (71.1%) reporting no previous IPV or DV experience than their overall percentage in the school population (62.4%), which was not statistically significant, *p* = 0.1229.

The majority of respondents (64%) indicated no previous IPV-specific education, with a minority of students (36%) reporting some previous educational experience. No temporal changes were noted over time, as each cohort was evaluated separately (C1, 36%; C2, 39%; C3 36%, *p* = 0.411). Note: The percentage of males and females reporting previous IPV education was different. Although males represented 62.4% of students, 71.1% of the No previous IPV education responses were from males, which was not statistically significant (χ^2^ = 2.380, d.f. = 1, *p* = 0.1229).

**Figure 2:**
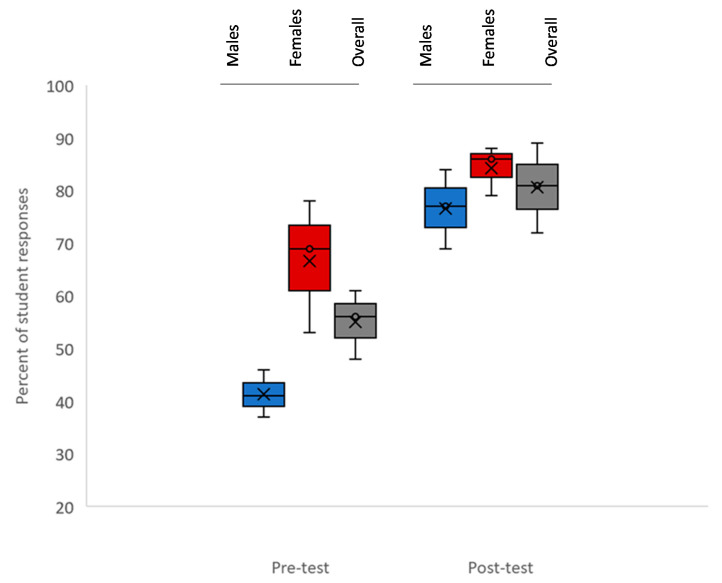
Percent of student responses indicating that vomestic violence (DV) or interpersonal violence (IPV) is a healthcare or dental issue (pre- and post). More than half of respondents in the pre-survey (*n* = 119/232 or 51.3%) indicated that IPV or DV was a healthcare or dental issue, which was initially higher among females (average 69%) than males (41%), *p* = 0.041. Overall percentages of students who felt DV/IPV was a healthcare or dental issue rose to 81% in the post-test, with responses among males (77%) and females (86%) more closely aligned, *p* = 0.221.

The second question sought to assess whether dental students perceived IPV or DV as a healthcare or dental healthcare issue (Figure 2). These data demonstrated that slightly more than half of all respondents in the pre-survey (*n* = 119/232 or 51.3%) indicated that IPV or DV was a healthcare or dental issue. However, more in-depth analysis revealed that responses from females (average 69%) were significantly higher than responses from males (41%) in the pre-test, *p* = 0.041. Following the educational seminar, the overall percentages of students who felt DV/IPV was a healthcare or dental issue rose to 81%. The responses among males (77%) and females (86%) were more closely aligned and were not significantly different, *p* = 0.221.

**Figure 3:**
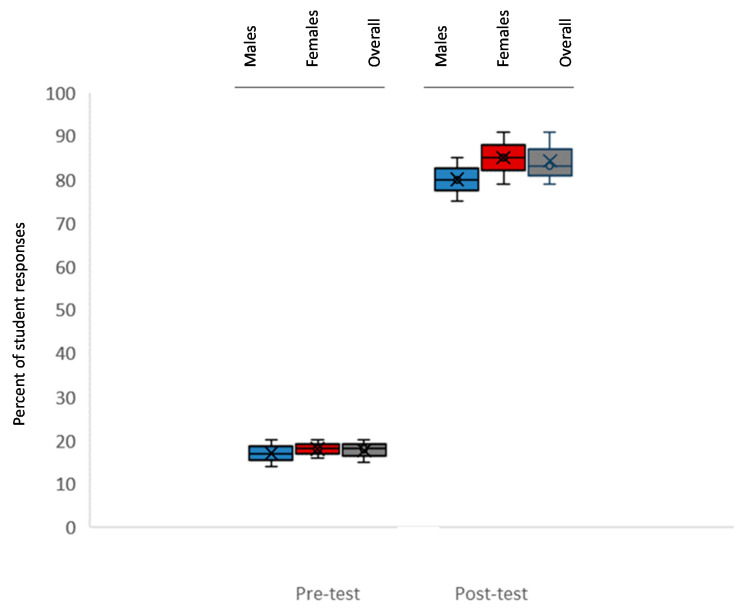
Percent of student responses indicating awareness of domestic violence (DV) or interpersonal violence (IPV) resources or referral information (pre- and post). Most initial responses (pre-test) indicated students were unaware of specific resources or referral (Average 18.1%), which were not sex-specific, *p* = 0.812. However, following the educational seminar, students who could identify the resources and referral information rose to 83%, which did not vary significantly between females and males, *p* = 0.566.

The last question sought to determine the percentage of students with awareness of resources and referral information for DV or IPV (Figure 3). The data analysis revealed that the vast majority of initial responses (pre-test) indicated students were unaware of specific resources, referral procedures, or other pertinent materials specific to Nevada or UNLV (Average 18.1%). A detailed analysis by sex revealed these deficiencies were not sex-specific, which may suggest that any previous educational experience may not have included resources, or that it was not provided in a local setting (e.g., UNLV), *p* = 0.812. However, following the educational seminar, the overall percentage of students who responded that they felt they knew the process for referral and could identify the resources and referral information rose to 83%, which did not vary significantly between females and males, *p* = 0.566.

## 4. Discussion

Based upon the limited number of previous studies available in this area, the objective of this study was to conduct a longitudinal assessment of knowledge, awareness, and beliefs regarding IPV among dental students in their first year of education to determine whether the results from the initial study from this group were representative of these responses [11]. This study was able to successfully analyze three additional years of dental student responses, which increases the strength of inferences that can be drawn from this type of longitudinal analysis [26,27]. In addition, this study also provides additional evidence that this type of educational intervention may be sufficient for implementation in healthcare settings to increase knowledge and awareness of graduate and professional students regarding resources and referrals for patients with DV or IPV needs—an important objective for most programs that strive to improve screening and support services [28,29].

This study also demonstrated significant differences in the pre-survey between male and female perceptions regarding whether DV and IPV are healthcare issues, which may reflect the findings of other students among medical and nursing graduates and professional students [30,31,32]. It is critically important that healthcare curricula address the differences in perception and education of all students with regard to DV and IPV, particularly when it comes to the differences and gaps between awareness and recognition among males and females and how these differences may disproportionately affect decisions to provide referrals and support to patients in need [33,34,35].

Despite the lack of awareness and knowledge among some females in the pre-survey group regarding DV and IPV, another significant finding from this study was that the overwhelming majority of students (including female students) did not appear to have adequate knowledge of resources or specific referral services [36,37]. More data regarding this overall lack of knowledge and whether this is commonly found among graduate and professional healthcare students may help to facilitate discussions regarding the curricular importance of including DV and IPV instruction in foundational healthcare educational instruction, particularly among dentists and other oral healthcare providers [38,39,40].

Although these findings provide a significant advance in our longitudinal assessment of dental student knowledge and awareness of DV and IPV within the oral healthcare setting, there are some limitations which should also be considered. For example, this study was completed in only one dental-school specific setting and may therefore not be representative of many graduate and professional healthcare students; however, limited information is available to make these comparisons [41,42]. In addition, no long-term follow up was completed to assess whether the knowledge and awareness of resources and referrals might be retained for use in subsequent years when more intensive clinical training occurs; however, other studies have shown some preliminary data regarding long-term retention of closely related training topics among medical and other healthcare students [43].

As more evidence accumulates that demonstrates many of the skills and competencies carried into professional practice are first developed in the educational setting, reviews, assessments, and feedback of preclinical dental education regarding important, but often overlooked, healthcare issues such as DV and IPV become more imperative [44,45]. The function of educational research in the assessment and evaluation of this type of training in the context of public health, particularly for healthcare students with areas of specialty that may be uniquely suited to finding, reporting, treating, and referring patients at risk for DV and IPV, has also become evident [46,47,48].

Although recent data may be lacking due to the interruption of routine work practices and healthcare visits due to the COVID-19 pandemic, some studies have suggested that the incidence and prevalence of DV and IPV may be increasing in some areas, including Nevada [49,50]. These data support the official position statement of the American College of Preventive Medicine that IPV and DV are important sociomedical problems that deserve professional, academic, and curricular attention to help provide capable, competent, and engaged healthcare providers with sufficient knowledge and awareness, not only of this issue, but of the resources and referrals that may help provide care and support for patients experiencing DV or IPV [51].

## 5. Conclusions

Although this study provides a longitudinal assessment of dental student knowledge and awareness of DV and IPV, this was institution-specific and was not performed in multiple sites to determine whether this is more broadly generalizable. In addition, the cross sectional (one-time sampling) nature of this study does not allow for an assessment of long-term information retention. It is hoped that future studies based upon these results will help to broaden and strengthen these findings and will incorporate these features to provide more in-depth and robust data to support these conclusions. However, the results of this study strongly suggest that targeted, information-specific educational seminars incorporated into a healthcare curriculum may be sufficient to provide dental students with an understanding of the key issues regarding IPV. Moreover, these trainings may be sufficient to increase student awareness of available resources and referrals, which may be of specific use when diagnosing and treating patients experiencing DV or IPV. With the acquisition of this type of specific knowledge and training, these students and future clinicians may be able to better provide specific information about resources and referrals for services to their patients who may be experiencing the adverse effects of DV or IPV.

## Figures and Tables

**Table 1:** Study participants.

	Survey Responses	Cohort	Statistical Analysis
**Sex**			
Females	*n* = 85 (36.6%)	*n* = 91 (37.6%)	Χ^2^ = 0.042, d.f. = 1
Males	*n* = 145 (62.5%)	*n* = 151 (62.4%)	*p* = 0.8368
No response	*n* = 2 (0.9%)		
**Race/ethnicity**			
White	*n* = 117 (50.4%)	*n* = 137 (56.6%)	Χ^2^ = 1.461, d.f. = 1
non-White (Minority)	*n* = 103 (44.4%)	*n* = 105 (43.4%)	*p* = 0.2268
Asian/Pacific Islander	*n* = 72 (31.0%)	*n* = 72 (29.8%)	
Hispanic	*n* = 22 (9.5%)	*n* = 25 (10.3%)	
Black/African Amer.	*n* = 5 (2.2%)	*n* = 6 (2.5%)	
No response	*n* = 12 (5.2%)		
**Age**			
Under 25 years	*n* = 88 (37.9%)	*n* = 99 (40.9%)	Χ^2^ = 0.372, d.f. = 1
Over 25 years	*n* = 144 (62.1%)	*n* = 143 (59.1%)	*p* = 0.5419

## Data Availability

The data presented in this study are available on request from the corresponding author. The data are not publicly available due to the study protocol data protection parameters requested by the IRB and OPRS for the initial study approval.
